# Ephrins in astrocytes: synaptic erasers on stage

**DOI:** 10.18632/oncotarget.14237

**Published:** 2016-12-26

**Authors:** Barbara Di Benedetto

**Affiliations:** ^1^ Department of Psychiatry and Psychotherapy, University of Regensburg, Regensburg, Germany; Regensburg Centre of Neuroscience, University of Regensburg, Regensburg, Germany

**Keywords:** astrocytes, ephrinA/EphA system, antidepressants, desipramine, neuropsychiatric disorders, Neuroscience

**Figure 1:**
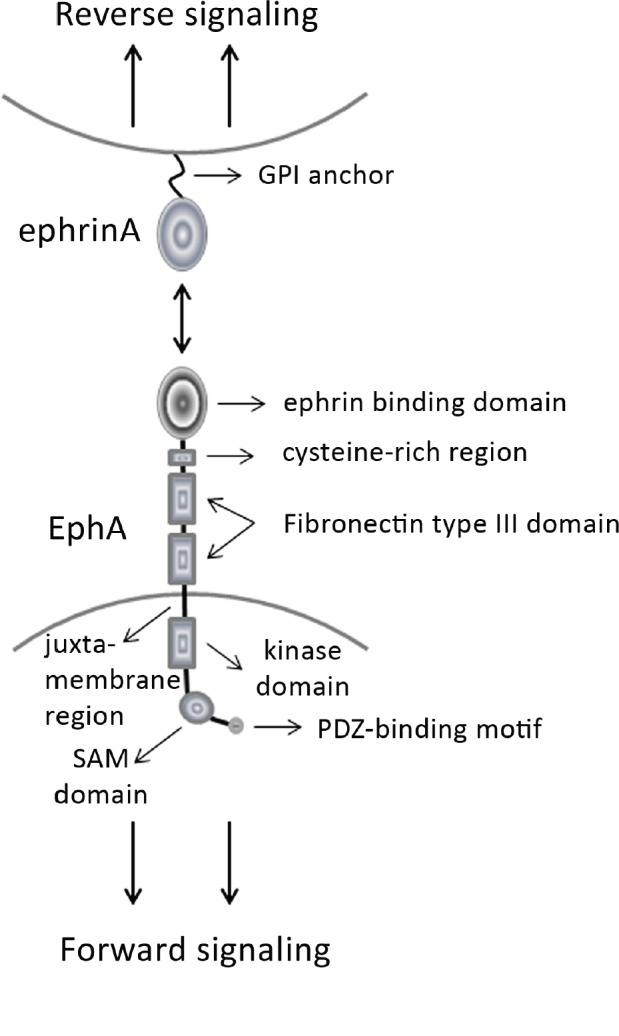
Schematic drawing of the ephrin/EphA system The bidirectional cell-to-cell communication character (“forward” and “reverse” signaling) is a typical feature of the ephrin/Eph system to execute a broader variety of functions. GPI, Glycosylphosphatidylinositol; SAM, sterile alpha motif; PDZ, Post-synaptic density protein 95 (PSD-95), Drosophila disc large tumor suppressor (Dlg1) and Zona occludens 1 (ZO- 1).

Among glia cells, astrocytes actively regulate the shaping and functions of the “tripartite synapse” [1]. Changes in synapses may influence diverse biological processes including long-term memory or the shift from acute to chronic pain, which can be dysfunctional in neuropsychiatric disorders such as major depressive disorder (MDD) or in neuropathic pain (NP), respectively [2]. Long-term potentiation (LTP) is considered a major cellular component of memory storage and relies on stabilization of synaptic changes dependent on the activation of several signalling pathways such as the Extracellular signal-Regulated Kinase/Mitogen Activated Protein Kinase (ERK/MAPK) pathway [3]. However, regional and cell-type specific localizations of ERK activity after LTP induction had never been characterized. The ERK pathway is also altered in animal models of depression and decreased levels of its downstream effectors ERK1 and ERK2 characterize post-mortem brains of depressive patients [4], indicating the high biomedical relevance of its pharmacological targeting to reverse aberrant behavioral phenotypes. Combining electrophysiology and immunohistochemistry, we showed that ERK signalling was increased in the *stratum radiatum*, but not in the *stratum pyramidale*, of the CA1 region of the hippocampus after LTP induction and a short-term administration of the antidepressant desipramine (DMI) to acute brain slices inhibited ERK activation and attenuated LTP. Additionally, ERK activity was peaking exclusively in astrocytes, but not in neurons of neither *stratum,* and this activation was prevented by DMI administration [2]. Simultaneously, transcription of the activity-dependent immediate early-gene (IEG) *Arc/ Arg 3.1 (Activity-regulated cytoskeleton-associated gene/ Activity regulated gene 3.1)* was enhanced in neurons of the *stratum pyramidale*, but its sustained activation was prevented by DMI treatment. As *Arc* is usually induced by ERK, but ERK was not affected neither by LTP induction nor by DMI in neurons, we investigated whether drug administration might have indirectly influenced neuronal *Arc* expression and LTP upon modulation of both the astrocytic ERK and the molecular glia/neuron interface in the *stratum radiatum*. A study by Filosa and colleagues had previously revealed that the ephrinA3/EphA4 receptor system, which acts as an essential bidirectional glia/ neuron communication mechanism, modulates several physiological processes including proper LTP induction and the shaping of neuronal synapses [5]. The EphA —> ephrinA activation is called “reverse signaling”, whereas the ephrinA —> EphA activation is called “forward signaling” (Figure 1). We postulated that its regulation by DMI treatment in the *stratum radiatum* of the CA1 might have been relevant in our experiments to induce an astrocyte-dependent synaptic remodeling during neuronal activity. Our findings indeed revealed that the administration of DMI to acute brain slices triggered alone an increased clustering of EphA4, an event usually correlated with enhanced EphA4 phosphorylation, synaptic plasticity and structural changes such as spine retraction. However, when we measured EphA4 phosphorylation after DMI treatment we did not observe any changes with respect to control slices. These results suggested a potential effect of DMI on predisposing neuronal circuits to be remodeled exclusively during neuronal activity. Unexpectedly, however, application of DMI concomitant to LTP induction reduced, and not increased, EphA4 clustering when compared to DMI alone, possibly indicating a decrease in synaptic contacts due to an EphA4-dependent spine retraction parallel to the observed LTP attenuation. These effects were reproduced by ephrinA3 administration which enhanced EphA4 phosphorylation as much as LTP induction enhanced EphA4 clustering, confirming a link between ephrinA3 activity and LTP. The effect was further reinforced in the presence of DMI, suggesting that an increased ephrinA3- dependent EphA4 phosphorylation upon DMI treatment may have triggered its internalization or spine retraction and clarified both the reduction in clustered EphA4 after LTP and the consequent LTP inhibition. This hypothesis was indeed confirmed by co-treatment of brain slices with DMI and SU6656, an inhibitor of EphA4 phosphorylation, which partially reversed effects of DMI on LTP [2].

Even though we restricted our analysis to the CA1 region of the hippocampus, we propose that such a mechanism of action of DMI could be extended to explain its therapeutic effects on NP [6]. Recent work has in fact showed that sciatic nerve ligation induces an astrocyte-dependent synapse formation in the somatosensory cortex and this event contributes to neuropathic mechanical allodynia [7]. DMI may reverse or even prevent the development of neuropathic allodynia by acting on the peripheral or cortical astrocyte ephrin signaling system. This effect may either induce a synaptic remodelling in the case of an advanced disease state with excessive synaptic contacts or help to avoid the onset of an undesirable synaptic formation, if used as preventive treatment.

The exploration of alternative molecular targets which could help to develop more specific and efficacious drug treatments for a broader spectrum of disorders might be supported by the search of cell-type specific responses to either beneficial or detrimental environmental stimuli as well as to pharmacological treatments with currently available therapeutic drugs.

Our study shows that drugs such as antidepressants may be investigated for their targeting of glia cells to favour the “erasing” of synaptic contacts which might have become “wrongly” wired in neuropsychiatric disorders or excessively produced after detrimental stimuli as is the case in NP. A further comparison of drug effects on an astrocyte-dependent synaptic remodelling in both healthy and diseased brains may help to identify novel targets to boost drug discovery. Additionally, our study suggests the ephrinA/EphA signaling system as a putatively deregulated candidate at the astrocyte-neuron interface in MDD or NP which may be targeted to reverse disease phenotypes and develop more efficacious therapeutic treatments.
